# Spatiotemporal Gait Variables and Step-to-Step Variability in Preschool-Aged Children Born Very Preterm at Risk for Developmental Coordination Disorder: A Cohort Study

**DOI:** 10.3390/children12091261

**Published:** 2025-09-19

**Authors:** Reem A. Albesher, Jennifer L. McGinley, Fiona L. Dobson, Benjamin F. Mentiplay, Tara L. FitzGerald, Kate L. Cameron, Jeanie L. Y. Cheong, Alicia J. Spittle

**Affiliations:** 1 Department of Rehabilitation Sciences, College of Health and Rehabilitation Sciences, Princess Nourah bint Abdulrahman University, Riyadh 11671, Saudi Arabia; 2 Department of Physiotherapy, University of Melbourne, Melbourne 3010, Australia; mcginley@unimelb.edu.au (J.L.M.); fdobson@unimelb.edu.au (F.L.D.); tara.fitzgerald@unimelb.edu.au (T.L.F.); k.cameron@unimelb.edu.au (K.L.C.); aspittle@unimelb.edu.au (A.J.S.); 3 Sport and Exercise Science, School of Allied Health, Human Services and Sport, La Trobe University, Melbourne 3086, Australia; b.mentiplay@latrobe.edu.au; 4 La Trobe Sport and Exercise Medicine Research Centre, School of Allied Health, Human Services and Sport, La Trobe University, Melbourne 3086, Australia; 5 Victorian Infant Brain Studies, Clinical Sciences, Murdoch Children’s Research Institute, The Royal Children’s Hospital, Flemington Rd, Melbourne 3052, Australia; jeanie.cheong@thewomens.org.au; 6 Department of Obstetrics, Gynaecology, and Newborn Health, University of Melbourne, The Royal Women’s Hospital, Melbourne 3010, Australia; 7 Department of Paediatrics, University of Melbourne, Melbourne 3010, Australia; 8 Neonatal Research, The Royal Women’s Hospital, Melbourne 3052, Australia

**Keywords:** developmental coordination disorder, preterm birth, gait variability, dual-task paradigm, motor impairment

## Abstract

Background/Objective: The gait pattern of children born very preterm shows gait decrements compared to their full-term peers in dual-task walking. It is essential to identify children at a higher risk for these gait deficits. The aim of this study was to compare spatiotemporal gait variables in preschool-age children born very preterm at risk for developmental coordination disorder (DCD) with those not at risk. Methods: Preschool-age children born < 30 weeks’ gestation. Risk for DCD was defined as (i) ≤16th percentile on the Movement Assessment Battery for Children—Second Edition, (ii) ≥80 on the Wechsler Preschool and Primary Scale of Intelligence-Fourth Edition, and (iii) without cerebral palsy. Spatiotemporal gait variables and variability were assessed using GAITRite^®^ during preferred speed, cognitive and motor dual-task, and tandem conditions. Variables included speed (cm/s), step time (s), cadence (steps/min), step length (cm), base of support (BOS; cm), and single and double support time (%gait cycle). Results: Of 111 children who were assessed, 26 children were classified as at risk for DCD. Most gait variables were similar between groups at preferred speed walking. Children at risk for DCD had wider BOS and shorter single support time in motor dual-tasking (mean difference [MD] = 0.86 cm, 95% confidence interval [CI] 0.10, 1.61; MD = −1.77%, 95% CI −3.36, −0.19) compared to those not at risk. Similarly, wider BOS and higher cadence were found when tandem walking (MD = 0.63 cm, 95% CI 0.07, 1.20; MD = 0.63 steps/min, 95% CI 0.07, 1.20). Conclusions: Children born very preterm at risk for DCD had poorer walking performance than those not at risk for DCD at preschool age, especially during dual-task situations. Clinicians may incorporate complex gait assessments into early evaluations to detect subtle impairments in children. Future research is needed to investigate the impact of gait variability on children’s daily lives and participation in sports activities.

## 1. Introduction

Despite advances in neonatal care, children born very preterm (VP; <32 weeks’ gestation) still face a high risk of subtle motor impairments [1,2]. Some research suggests that VP children tend to develop near-typical gait patterns at their preferred speed during early childhood [3,4]. However, emerging evidence shows that gait deficits become apparent in more challenging walking conditions, such as dual-task or tandem walking [4]. For example, children born VP display differences in their base of support (BOS); with a broader BOS during motor or cognitive dual-tasks and tandem walking, and increased variability in BOS at preferred speed and during tandem walking compared to their term-born peers [4]. Limited research has examined which VP children are at the greatest risk for persistent gait deficits. Therefore, identifying high-risk subgroups within the VP population with gait impairment may help address their needs through targeted assessments and interventions.

One explanation for the differences in gait patterns of children born VP compared with their term peers could be that they exhibit higher rates of motor impairment [2]. These motor impairments can range from developmental coordination disorder (DCD) to cerebral palsy (CP) [1,2]. It is crucial to target research, including gait investigation, of children born preterm without major neurological impairment, as mild to moderate motor impairment often remains undiagnosed until school age, potentially limiting timely support [5]. DCD is a common motor impairment in children born VP, with an incidence up to eight times higher in children born VP than in those born at term, when using a cut-off of <16th centile on the Movement Assessment Battery for Children (MABC) [6]. DCD is characterised by a substantial impairment in motor coordination that interferes with activities of daily living [7]. The gait patterns of children with DCD have been described in the literature as “awkward”, “slow”, and “clumsy” [8]. There is little evidence of spatiotemporal differences in children with DCD compared with typically developing children when walking at a preferred speed [9]. A recent study by Goetschalck et al. (2024) showed that school-age children with DCD walk with higher spatiotemporal variability compared to typically developing controls [10]. Further, greater gait decrements were seen in children with DCD than in typically developing children when performing a motor dual-task, but this did not apply to the cognitive dual-task [11]. Furthermore, younger children with DCD show greater variability than their older peers [12]. These findings suggest that task complexity reveals gait deficits not visible during simple walking, emphasising a gap in understanding how early gait variability appears in VP children at risk of DCD.

A formal diagnosis of DCD is not recommended until five years of age [13]. However, emerging evidence suggests that children at high risk for DCD, such as those born very preterm, can be identified before starting school using early detection methods [14,15]. Preschool age is a crucial period for developing functional skills, marked by significant advances in locomotion and nervous system development [16]. Additionally, children at this age encounter more balance and gait challenges when performing concurrent tasks compared to their older peers [17]. Therefore, preschool years form the foundation for school readiness and participation in organised sports, where children face greater interactions and challenges. Detecting children at risk of gait challenges early enables timely referrals and interventions before they begin school. These findings underscore the significance of early detection in preschool-aged children with greater gait variability and gait decrements, particularly in those born with VP. This may help provide targeted therapies that focus on gait variability and difficulties, which can enhance motor skills, reduce fall risk, and increase participation in daily activities at school, on playgrounds, and during sports before transition to school.

There is a limited understanding of gait variability and its impact in children born VP, and identifying those at greater risk for greater gait variability and decrements. The aim of this study was to compare spatiotemporal gait variables and step-to-step variability in 4- to 5-year-old children born < 30 weeks’ gestation, classified as at risk for DCD, with those not at risk for DCD at preferred speed, cognitive dual-task, motor dual-task, and tandem walking conditions. We hypothesised that VP children at risk for DCD would show greater gait variability during dual-task and tandem walking conditions compared with VP children not at risk for DCD.

## 2. Materials and Methods

### 2.1. Participants

Children between 4 and 5 years of age who were included in the Victorian Infants Brain Study longitudinal cohort study (VIBeS) of Infants born < 30 weeks’ gestation (referred to as VP) were recruited initially at birth from the Royal Women’s Hospital, Melbourne, Australia, between January 2011 and December 2013 [18]. Infants with congenital abnormalities known to affect development or born to non-English speaking parents were excluded, as no funding was available for interpreters. One hundred forty-nine children born VP were recruited at birth; six children died in the neonatal period, and one was diagnosed with Down’s Syndrome and was later excluded.

### 2.2. Predictor

Children were seen between 4 and 5 years’ corrected age (CA) for neurodevelopmental follow-up, including gait assessment at The Royal Children’s Hospital, Melbourne, Australia [18]. Ethical approval was obtained from the Royal Women’s Hospital and the Royal Children’s Hospital Human Research Ethics Committees [18]. Caregivers provided written informed consent on behalf of their child. Children’s age was corrected for prematurity, as chronological age results in underestimating the performance of children born preterm [19]. Assessments were completed by trained assessors blinded to the child’s medical history and previous evaluations. Height, weight, and leg length were measured prior to gait assessment.

#### 2.2.1. Risk for Developmental Coordination Disorder

Children were defined as at risk for DCD using three criteria: (i) Movement Assessment Battery for Children-Second Edition (MABC-2) score ≤ 16th percentile, (ii) full-scale composite intelligence quotient (IQ) of ≥80 on the Wechsler Preschool and Primary Scale of Intelligence-Fourth Edition (WPPSI-IV), and (iii) no diagnosis of CP [20,21]. The classification of being at risk for DCD was used because a diagnosis of DCD is only recommended after five years of age [13]. Children who scored > 16th percentile on the MABC-2 and without CP were classified as not at risk for DCD.

**Movement Assessment Battery for Children, Second Edition**

The MABC-2 is valid and reliable for children aged 3 to 16 years, including children born preterm [21,22]. The MABC-2 is the most common assessment tool to identify children at risk for motor impairment, with a cut-off score ≤ 16th percentile considered as ‘at risk of motor impairment’ and ≤5th percentile as with ‘significant motor impairment’ [20,21]. Children who could not complete the assessment due to motor impairment scored the lowest possible standard score [1] and a percentile (0.1). Missing scores due to behavioural or cognitive impairment were treated as missing data.

**Wechsler Preschool and Primary Scale of Intelligence, 4th Edition**

The WPPSI-IV is one of the primary instruments to evaluate cognitive ability. The full-scale IQ composite score of the Australian and New Zealand Standardised Edition was used in this study [23]. A full-scale IQ composite score ≥ 80 on WPPSI-IV was chosen as a cut-off, as children who score under 80 are classified as borderline on the WIPPSI-IV.

#### 2.2.2. Gait Assessment

Spatiotemporal gait variables were measured using the GAITRite^®^ electronic walkway system (CIR Systems Inc., Clifton, NJ, USA). The GAITRite^®^ system is a valid and reliable assessment system that consists of a 16-foot-long electronic mat with 23,040 integrated pressure sensors. The use of the GAITRite^®^ to assess the gait patterns of typically developing children and children with motor impairments is well established [24,25]. Children walked across the walkway with two metres added to each end to avoid acceleration and deceleration effects. Four barefoot walking conditions were assessed consecutively: preferred speed, cognitive dual-task, motor dual-task, and tandem walking. Before each walking condition, children were given a verbal and visual demonstration, along with one practice trial to familiarise them with the procedure. Children first walked at their preferred speed without any concurrent task. Next, children were instructed to walk at their preferred speed while simultaneously performing one of the concurrent tasks. Concurrent tasks were (i) cognitive; naming as many items as possible per pre-set categories, measured as the number of accurate verbal responses per trial and (ii) motor; balancing four table tennis balls on a 20 cm plate, measured as the number of balls dropped per trial. Finally, children were instructed to walk along a straight, non-slip 5 cm wide line placed along the walkway for the tandem walking condition. Children were not instructed to walk only in the heel-to-toe pattern or any other pattern. Further, children were instructed to return to the line and continue walking if they lost balance and stepped outside the taped line. Further details of the gait assessment are previously published in Albesher et al., 2022 [4].

Six successful walking trials were captured for each condition where possible. Trials were excluded if the child walked off the electronic mat, was identified as walking atypically by the parents, or had fewer than six consecutive steps. A detailed gait assessment and data processing protocol were used, and all assessed data were reviewed by a single assessor (RA) to ensure consistency of inclusion and processing.

Each walking trial was analysed using GAITRite^®^ Platinum, and then all gait variables were averaged per condition. The spatiotemporal gait variables evaluated included: speed (cm/s), step time (s), cadence (steps/min), step length (cm), base of support (BOS; cm), single support time (%gait cycle) and double support time (%gait cycle). Step-to-step variability was calculated as the standard deviation of all steps recorded per participant from all successful trials per condition. Gait variability was assessed for step time (s), step length (cm), BOS (cm), single support time (s) and double support time (s).

### 2.3. Statistical Analysis

Data were analysed using Stata 16.0 (StataCorp, College Station, TX, USA). The gait data distribution was inspected before analysis, and log transformation was used for gait variables that were not normally distributed. Gait data extracted from the GAITRite for both sides of the body were all incorporated into this analysis.

Linear regression models were used to assess the relationships between risk for DCD and gait variables (spatiotemporal variables and their variability) across the four walking conditions, adjusted for lower-limb length [4,26]. For the concurrent task performance, univariable linear regression models were used to investigate associations between risk for DCD and cognitive and motor dual-task scores. Each regression model was fitted with generalised estimating equations to allow for clustering among multiple births within a family.

The minimal clinically important difference (MCID) is typically described as 0.5 standard deviations, indicating a moderate effect size. Using 0.5 SD as a distribution-based estimate is widely recognised in clinical and rehabilitation research as representing a moderate effect size and a reasonable approximation of clinical significance [27,28]. The MCID was estimated for each spatiotemporal variable in all four walking conditions as 0.5 SD of the pooled values of preschool-aged children born VP [27]. Pooled data from preschool-aged children born VP, including the current sample, were published earlier [4].

## 3. Results

**Figure 1:**
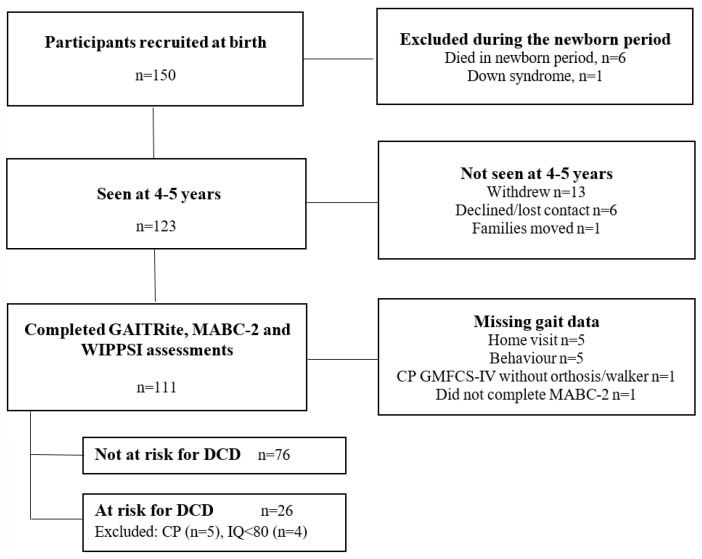
The flow of study participants through the study. CP, cerebral palsy; DCD, Developmental Coordination Disorder; GMFCS, Gross Motor Function Classification System; MABC-2; Motor Assessment Battery for Children, 2nd Edition; n, number; WPPSI, Wechsler Preschool and Primary Scale of Intelligence, Fourth Edition.

Of the 143 infants recruited at birth for the VIBeS cohort, 123 children born VP were seen at 4–5 years’ CA for follow-up, with 111 children (90.24%) completing both gait and motor competence assessments. There were 35/111 children (31.53%) who had a MABC-2 score ≤ 16th percentile, however, children with CP (n = 5; 14.29%) or with a full-scale IQ of <80 (n = 4; 11.43%) were excluded, resulting in 26/111 children (23.42%) classified as at risk for DCD (Figure 1). Not all children completed six successful walking trials in each condition (e.g., due to behaviour, fatigue, etc.). Participants’ characteristics and the number of participants who completed each condition are presented in Table 1.

The associations between being at risk for DCD and spatiotemporal gait variables are presented in Table 2, and the associations between being at risk for DCD and step-to-step variability of spatiotemporal variables are shown in Table 3. In the preferred speed condition, being at risk for DCD was associated with greater variability in BOS (mean difference [MD] = 0.51 cm, 95% confidence interval [CI] 0.27 to 0.75, *p* < 0.001), with minimal evidence for associations with other spatiotemporal gait variables or variability. During dual-task walking, being at risk for DCD was associated with greater variability in step length and BOS during the cognitive dual-task condition. Being at risk for DCD was also associated with a wider BOS, decreased single support time, and increased double support time, as well as higher variability in step time, step length and single support time in the motor dual-task condition. Further, being at risk for DCD was associated with higher cadence, shorter step time, wider BOS and more variable BOS in tandem walking. All mean differences meet the MCID (0.5 SD of pooled VP gait data) except double support time in the motor dual-task condition and BOS variability in the tandem condition.

There was minimal evidence that being at risk for DCD was associated with a difference in performance in the concurrent task of a dual-task condition, i.e., the number of accurate verbal responses in the cognitive dual-task condition (MD = −0.30 words, 95% CI −0.78 to 0.17, *p* = 0.213) and the number of balls dropped in the motor dual-task condition (MD = 0.28 balls, 95% CI −0.33 to 0.89, *p* = 0.367).

## 4. Discussion

This study found that children born VP at risk for DCD had greater challenges with walking performance in the dual-task paradigm and tandem conditions than children born VP not at risk for DCD at 4–5 years’ CA. Children at risk for DCD walked with comparable gait patterns to children not at risk at their preferred speed, with only greater BOS variability. However, walking in more challenging dual-task and tandem conditions resulted in more gait impairments and higher variability in children at risk for DCD, though they had a similar performance in the concurrent task, to those not at risk for DCD.

The clinical meaningfulness of the differences in characteristics between children born VP at risk and not at risk for DCD has not been directly investigated; however, group differences found in this study were comparable in magnitude to known minimal clinically important differences for the variables reported in the previous investigation of gait variables of the current cohort of VP children [4], with the exception of double support time in the motor dual-task condition and BOS variability in the tandem condition. This suggests that most gait differences found in this study may be meaningful. A single difference may not necessarily indicate a meaningful difference for the child or their family [29]. Therefore, we recommend considering the differences in the gait pattern as a whole. Furthermore, the clinical implications of the study findings need to be considered with caution, as they were not directly tested in this study and may not necessarily indicate a meaningful difference for the child or their family [29].

Walking freely at a preferred speed was found to be not a particularly challenging activity for preschool-age children at risk for DCD. The comparable gait pattern in preferred speed between children born VP at or not at risk for DCD is in broad agreement with an earlier systematic review that reported a similar pattern of comparable gait at preferred walking in school-age children with DCD [9]. However, children at risk for DCD did not show variation from their non-at-risk peers in gait variability except for BOS. Similarly, earlier evidence shows that children born VP walk with a comparable gait pattern to term-born children in preferred speed walking at preschool age [4] and school age [30].

Our findings add to the current knowledge about the gait of children born VP by identifying a vulnerable subgroup at greater risk of gait difficulties. Our results support previous research indicating that children at risk for DCD are more susceptible to dual-task gait decrements [11]. Furthermore, our findings suggest that motor dual-task walking might be more challenging than cognitive dual-task walking, as per previous studies in children with DCD [11,31] and typically developing children [17]. The notion that motor dual-task results in greater impairments is supported by the multiple resources theory [32]. This theory suggests that processing may require several resources and that two tasks will interfere if they share common resources [33]. Therefore, walking while performing a concurrent motor task—which shares the same resources as walking—can result in greater gait decrements than a concurrent cognitive task [33].

Our research found a more variable gait pattern in children at risk for DCD than in those not at risk for DCD in dual-task and tandem conditions. An increased gait variability reflects a more irregular stepping pattern during walking [34]; our findings indicate a less regular and automated gait pattern in preschool-aged children born VP at risk for DCD than those not at risk. Our finding is in line with the reported increased variability in older children with DCD [10]. Gait variability is less examined in young children compared to older adults, where increased gait variability has been associated with a higher risk of falling in the elderly population [34]. Gait variability may have important functional implications for children; however, it is yet to be intensively investigated. Step-to-step variability describes the fluctuations in gait measures from one step to the next [30]. This variability of spatiotemporal variables is thought to reflect the automaticity and consistency of walking, with variability decreasing as children grow older [35,36]. Preschool-age children experience greater difficulty with balance and gait while engaging in simultaneous tasks compared to older children [17]. These years lay the ground for school readiness and involvement in organised sports, where children are exposed to more interactions and challenges. Gait variability tends to be higher in children with developmental and motor disorders—such as CP, traumatic brain injuries, autism spectrum disorder, and DCD—compared to typically developing children walking at their preferred speed [37,38,39,40,41,42]. The higher gait variability in our cohort of children at risk for DCD may match the general description of walking performance in children at risk for DCD as clumsy and awkward, with frequent falls [8].

While DCD is not formally diagnosed until later in childhood [13], early detection of children at risk for DCD is possible and desirable for effective early intervention [16,17]. There is promising evidence that early intervention is beneficial for young children with or at risk for DCD [43]. Further, there is strong evidence for the effect of early developmental intervention programmes on the motor outcomes in infants born preterm who are at high risk for DCD [44].

The strengths of this study include the prospective recruitment of the participants. We included children born VP without motor impairment as a control group in this study to examine the differences within the VP population and identify the more vulnerable group, thereby providing an opportunity for more targeted developmental monitoring. Furthermore, gait was assessed in a variety of walking conditions that mimic aspects of walking challenges that occur in children’s daily lives.

There were also some limitations to our study. Walking conditions were not randomised, potentially introducing order effects. The difficulty of the concurrent tasks was not assessed in isolation, which may have affected task difficulty and comparability. Additionally, not all children completed a gait assessment due to fatigue, behavioural issues, or inability during home visits, and the small sample of children at risk for DCD might limit the detection of meaningful changes. Furthermore, the study is limited by the relatively low precision of some findings, which increases uncertainty in effect estimates, and by not adjusting for multiple comparisons, which may elevate the risk of Type I error.

This study attempted to identify high-risk subgroups within the VP population, i.e., those at risk for DCD. Our results highlight the importance of considering performance in different walking conditions and concurrent tasks when assessing children’s gait and planning intervention. Identifying preschool-aged children at risk of a formal DCD diagnosis later in childhood can facilitate early screening, intervention, and referrals. Knowing the effects of dual-task on the walking performance of children with or at risk of non-CP motor impairment may raise awareness to minimise the impact on gait and task performance, and avoid accidental loss of balance, i.e., falls. This may involve modifying daily routine activities and sports as necessary, by making them either harder or easier. Follow-up of children at risk for DCD who may not necessarily display gross motor problems, but could be challenged by more difficult daily situations, is also essential.

Future research is needed to investigate how the reported walking performance in children born VP at risk for DCD impacts other tasks of children’s everyday lives and sports activities. Studies should also examine the effects of different types of concurrent tasks on the gait of children at risk for DCD. Similarly, researchers should consider investigating the asymmetry of gait variables in children born VP at risk for DCD. Future research is needed to assess whether perinatal or environmental factors may have affected the reported gait outcomes. Future research investigating dual-task performance may benefit from the use of randomised sequences, progressively more challenging concurrent tasks, and applying different strategies to tailor cognitive and motor tasks to individual abilities, such as baseline single-task measurement, adaptive task difficulty, relative task scaling, and task prioritisation instructions.

## 5. Conclusions

Our study found that 4–5-year-old children born VP at risk for DCD had greater walking challenges than children born VP not at risk for DCD. Children at risk for DCD are more vulnerable to the effects of an imposed concurrent task and tandem walking than their peers who are not at risk for DCD. This study highlights the importance of early identification and early intervention throughout the preschool period to support motor development in children born VP at risk for DCD and their families. Future research is needed to understand the association between the walking performance of children at risk for DCD and whether intervention can improve outcomes.

## Figures and Tables

**Table 1:** Characteristics of children born very preterm at risk or not for DCD.

Characteristics	At Risk for DCD n = 26	Not at Risk for DCD n = 76
PERINATAL CHARACTERISTICS		
Gestational age (weeks), mean (SD)	27.74 (1.57)	27.87 (1.33)
Birth weight (g), mean (SD)	1044.12 (264.93)	1045.55 (243.45)
Female sex, n (%)	11 (42.31%)	41 (53.95%)
Multiple birth, n (%)	9 (34.61)	33 (43.42%)
4–5 YEARS FOLLOW-UP		
CA at assessment (years), mean (SD)	4.63 (0.12)	4.67 (0.14)
Attending preschool, n (%)	25 (96.15%)	71 (93.42%)
Weight at assessment (kg), mean (SD)	17.45 (3.98)	17.44 (2.33)
Height at assessment (cm), mean (SD)	105.64 (5.25)	106.45 (4.40)
Leg length at assessment (cm), mean (SD)	55.04 (4.39)	55.37 (3.55)
MABC-2—Total standard score, mean (SD)	5.35 (1.45)	10.62 (2.05)
≤5th percentile on MABC-2, n (%)	12 (46%)	—
WPPSI—FSCIQ score, mean (SD)	96.35 (8.67)	105.33 (12.07)
Preferred speed condition, ^#^ n (%)	26 (100%)	75 (98.68%)
Cognitive dual-task condition, ^#^ n (%)	24 (92.31%)	68 (89.47%)
Motor dual-task condition, ^#^ n (%)	25 (96.15%)	72 (94.74%)
Tandem condition, ^#^ n (%)	24 (92.31%)	74 (97.39%)

^#^ number of participants who have any number of valid walking trials in the condition; CA, corrected age; cm, centimetres; DCD, Developmental Coordination Disorder; FSCIQ, Full-scale composite IQ score; kg, kilograms; MABC-2, Motor Assessment Battery for Children, 2nd Edition; n, number; SD, standard deviation; WPPSI—FSCIQ, Wechsler Preschool and Primary Scale of Intelligence—Full scale IQ.

**Table 2:** Mean differences in spatiotemporal gait variables and concurrent task performance between children born very preterm at risk for DCD and those not at risk in preferred speed, dual-task paradigm, and tandem walking at 4–5 years’ corrected age.

Gait Variable	Walking Condition	At Risk for DCD Mean (SD)	Not at Risk for DCD Mean (SD)	Mean Difference (95% CI)	*p* Value
**Speed (cm/sec)**	*Preferred speed*	99.43 (17.79)	100.65 (18.07)	−0.65 (−10.46, 9.15)	0.896
	*Dual-task-Cognitive*	70.35 (21.13)	72.14 (19.27)	0.20 (−11.40, 11.80)	0.973
	*Dual-task-Motor*	55.12 (25.04)	60.33 (18.33)	−5.40 (−14.74, 3.95)	0.258
	*Tandem walk*	69.22 (20.09)	65.67 (16.30)	4.40 (−4.80, 13.60)	0.348
**Cadence (steps/min)**	*Preferred speed*	145.17 (18.84)	144.86 (15.53)	−0.44 (−7.63, 6.74)	0.904
	*Dual-task-Cognitive*	122.84 (26.92)	122.89 (19.54)	1.08 (−10.51, 12.67)	0.856
	*Dual-task-Motor*	112.36 (27.73)	115.89 (20.12)	−3.13 (−12.95, 6.68)	0.532
	*Tandem walk*	128.12 (23.76)	117.71 (20.87)	10.93 (1.63, 20.23)	0.021 *^
**Step time (sec)**	*Preferred speed*	0.42 (0.05)	0.42 (0.05)	0.00 (−0.02, 0.02)	0.859
	*Dual-task-Cognitive*	0.51 (0.11)	0.50 (0.08)	0.00 (−0.05, 0.05)	0.869
	*Dual-task-Motor*	0.57 (0.15)	0.53 (0.10)	0.03 (−0.03, 0.08)	0.317
	*Tandem walk*	0.48 (0.09)	0.53 (0.11)	−0.05 (−0.09, −0.01)	0.023 *^
**Step length (cm)**	*Preferred speed*	40.97 (4.15)	41.53 (4.98)	0.09 (−2.92, 3.11)	0.952
	*Dual-task-Cognitive*	34.00 (5.47)	34.76 (4.96)	−0.24 (−3.66, 3.18)	0.892
	*Dual-task-Motor*	28.16 (6.82)	30.70 (5.23)	−2.41 (−5.51, 0.68)	0.127
	*Tandem walk*	31.97 (5.21)	33.20 (4.06)	−1.17 (−3.55, 1.22)	0.338
**Base of support (cm)**	*Preferred speed*	8.24 (1.38)	7.94 (1.86)	0.32 (−0.43, 1.08)	0.401
	*Dual-task-Cognitive*	9.73 (1.60)	9.52 (2.27)	0.41 (−0.60, 1.42)	0.423
	*Dual-task-Motor*	9.73 (1.37)	8.71 (1.69)	0.86 (0.10, 1.61)	0.027 *^
	*Tandem walk*	4.17 (1.57)	3.12 (0.94)	0.87 (0.18, 1.56)	0.013 *^
**Single limb support** **(%)**	*Preferred speed*	40.94 (1.85)	40.96 (1.60)	−0.01 (−0.69, 0.68)	0.985
	*Dual-task-Cognitive*	38.02 (2.35)	38.64 (2.48)	−0.38 (−1.48, 0.73)	0.505
	*Dual-task-Motor*	34.14 (4.27)	35.87 (3.42)	−1.77 (−3.36, −0.19)	0.028 *^
	*Tandem walk*	38.47 (2.68)	38.28 (2.75)	0.03 (−1.00, 1.06)	0.957
**Double limb support** **(%)**	*Preferred speed*	18.27 (3.17)	17.98 (2.78)	0.07 (−1.47, 1.61)	0.928
	*Dual-task-Cognitive*	24.33 (4.66)	23.03 (4.47)	0.48 (−1.85, 2.81)	0.686
	*Dual-task-Motor*	31.27 (8.26)	27.91 (6.27)	1.11 (1.01, 1.22) ^#^	0.036 *
	*Tandem walk*	22.72 (4.79)	22.94 (4.28)	0.07 (−1.99, 2.14)	0.944

^#^ Log transformed values have been retransformed back using the exponential function to give geometric mean ratios and associated 95% confidence intervals; * *p*-value < 0.05; ^ Mean difference meets the minimal clinically important difference in the pooled values of preschool-aged children born very preterm; CI, confidence interval; cm, centimetres; DCD, Developmental Coordination Disorder; min, minutes; sec, seconds.

**Table 3:** Mean differences in step-to-step variability between children born very preterm at risk for DCD and those not at risk in preferred speed, dual-task paradigm, and tandem walking at 4–5 years’ corrected age, adjusted for leg length.

Gait Variable	Walking Condition	At Risk for DCD Mean (SD)	Not at Risk for DCD Mean (SD)	Mean Difference (95% CI)	*p* Value
**Step time variability (sec)**	*Preferred speed*	0.04 (0.03)	0.04 (0.02)	1.04 (0.81, 1.33) ^#^	0.766
	*Dual-task-Cognitive*	0.08 (0.05)	0.07 (0.04)	1.01 (0.79, 1.52) ^#^	0.564
	*Dual-task-Motor*	0.10 (0.06)	0.08 (0.06)	1.33 (1.03, 1.71) ^#^	0.029 *^
	*Tandem walk*	0.09 (0.05)	0.10 (0.06)	−0.01 (−0.03, 0.01)	0.500
**Step length variability (cm)**	*Preferred speed*	4.17 (1.36)	3.75 (1.18)	0.31 (−0.20, 0.83)	0.229
	*Dual-task-Cognitive*	5.33 (1.36)	4.22 (1.32)	0.99 (0.21, 1.77)	0.013 *^
	*Dual-task-Motor*	4.79 (1.16)	4.39 (1.43)	0.63 (0.07, 1.20)	0.028 *^
	*Tandem walk*	5.72 (1.49)	5.18 (1.24)	0.53 (−0.146, 1.21)	0.125
**Base of support variability (cm)**	*Preferred speed*	2.93 (0.64)	2.41 (0.50)	0.51 (0.27, 0.75)	<0.001 *^
	*Dual-task-Cognitive*	2.98 (0.62)	2.55 (0.65)	0.32 (0.02, 0.63)	0.039 *^
	*Dual-task-Motor*	2.39 (0.43)	2.27 (0.50)	0.24 (−0.05, 0.53)	0.106
	*Tandem walk*	2.64 (0.70)	2.02 (0.53)	0.65 (0.12, 1.19)	0.017 *
**Single limb support time variability (sec)**	*Preferred speed*	0.04 (0.03)	0.03 (0.01)	1.08 (0.85, 1.37) ^#^	0.536
	*Dual-task-Cognitive*	0.06 (0.03)	0.05 (0.02)	0.01 (−0.00, 0.03)	0.197
	*Dual-task-Motor*	0.06 (0.03)	0.05 (0.02)	0.01 (0.00, 0.02)	0.005 *^
	*Tandem walk*	0.07 (0.03)	0.07 (0.03)	0.00 (−0.02, 0.014)	0.809
**Double limb support time variability (sec)**	*Preferred speed*	0.04 (0.02)	0.03 (0.01)	1.14 (1.09, 1.42) ^#^	0.234
	*Dual-task-Cognitive*	0.07 (0.04)	0.06 (0.06)	1.08 (0.78, 1.51) ^#^	0.633
	*Dual-task-Motor*	0.12 (0.09)	0.09 (0.09)	1.36 (0.96, 1.90) ^#^	0.079
	*Tandem walk*	0.08 (0.05)	0.09 (0.08)	0.83 (0.61,1.14) ^#^	0.250

^#^ Log transformed values have been retransformed back using the exponential function to give geometric mean ratios and associated 95% confidence intervals; * *p*-value < 0.05; ^ Mean difference meets the minimal clinically important difference in the pooled values of preschool-aged children born very preterm; CI, confidence interval; cm, centimetres; DCD, Developmental Coordination Disorder; sec, seconds.

## Data Availability

The data presented in this study are available upon request from the corresponding author. (The data are not publicly available.)
